# Effects of Cationic Microbubble Carrying CD/TK Double Suicide Gene and α_V_β_3_ Integrin Antibody in Human Hepatocellular Carcinoma HepG2 Cells

**DOI:** 10.1371/journal.pone.0158592

**Published:** 2016-07-08

**Authors:** Jiale Li, Ping Zhou, Lan Li, Yan Zhang, Yang Shao, Li Tang, Shuangming Tian

**Affiliations:** 1 Department of Ultrasound,the Third Xiangya Hospital, Central South University, Changsha, Hunan China; 2 MDFLOW System,Corporate Park of Doral, Doral, Florida, United States of America; University of Sassari, ITALY

## Abstract

**Objective:**

Hepatocellular carcinoma (HCC), mostly derived from hepatitis or cirrhosisis, is one of the most common types of liver cancer. T-cell mediated immune response elicited by CD/TK double suicide gene has shown a substantial antitumor effect in HCC. Integrin α_V_β_3_ over expresssion has been suggested to regulate the biology behavior of HCC. In this study, we investigated the strategy of incorporating CD/TK double suicide gene and anti-α_V_β_3_ integrin monoclonal antibodies into cationic microbubbles (CMBs_αvβ3_), and evaluated its killing effect in HCC cells.

**Methods:**

To improve the transfection efficiency of targeted CD/TK double suicide gene, we adopted cationic microbubbles (CMBs), a cationic delivery agent with enhanced DNA-carrying capacity. The ultrasound and high speed shearing method was used to prepare the non-targeting cationic microbubbles (CMBs). Using the biotin-avidin bridge method, α_V_β_3_ integrin antibody was conjugated to CMBs, and CMBs_αvβ3_ was generated to specifically target to HepG2 cells. The morphology and physicochemical properties of the CMBs_αvβ3_ was detected by optical microscope and zeta detector. The conjugation of plasmid and the antibody in CMBs_αvβ3_ were examined by immunofluorescent microscopy and flow cytometry. The binding capacities of CMBs_αvβ3_ and CMBs to HCC HepG2 and normal L-02 cells were compared using rosette formation assay. To detect EGFP fluorescence and examine the transfection efficiencies of CMBs_αvβ3_ and CMBs in HCC cells, fluorescence microscope and contrast-enhanced sonography were adopted. mRNA and protein level of CD/TK gene were detected by RT-PCR and Western blot, respectively. To evaluate the anti-tumor effect of CMBs_αvβ3_, HCC cells with CMBs_αvβ3_ were exposed to 5-flurocytosine / ganciclovir (5-FC/GCV). Then, cell cycle distribution after treatment were detected by PI staining and flow cytometry. Apoptotic cells death were detected by optical microscope and assessed by MTT assay and TUNEL-staining assay.

**Results:**

CMBs_αvβ3_ had a regular shape and good dispersion. Compared to CMBs, CMBs_αvβ3_ had more stable concentrations of α_V_β_3_ ligand and pEGFP-KDRP-CD/TK, and CMBs_αvβ3_ was much sticker to HepG2 HCC cells than normal liver L-02cells. Moreover, after exposed to anti-α_V_β_3_ monoclonal antibody, the adhesion of CMBs_αvβ3_ to HepG2 cells and L-02 cells were significantly reduced. Also, CMBs_αvβ3_ demonstrated a substantially higher efficiency in pEGFP-KDRP-CD/TK plasmid transfection in HepG2 cells than CMBs. In addition, CMBs_αvβ3_ could significantly facilitate 5-FC/GCV-induced cell cycle arrest in S phase. Moreover, treatment of 5-FC/GCV combined with CMBs_αvβ3_ resulted in a marked apoptotic cell death in HepG2 and SK-Herp-1 HCC cells. In vitro, treatment of 5-FC/GCV combined with CMBs_αvβ3_ suppresed cell proliferation. In nude mice model, 5-FU + GCV combined with plasmid + CMBs_αvβ3_were able to significantly suppress tumor volumes.

**Conclusion:**

Through biotin-avidin mediation system, CMBs_αvβ3_ were successfully generated to specifically target HCC HepG2 cells. More importantly, CMBs_αvβ3_ could significantly facilitate 5-FC/GCV-induced cell cycle arrest and apoptotic cell death in HepG2 cells. Our study demonstrated a potential strategy that could be translated clinically to improve liver tumor gene delivery.

## Introduction

Hepatocellular carcinoma (HCC), one of the most common malignant tumor with a high incidence and mortality in the world, threatens people’s life during past decades [1]. With the development of molecular biology and genetic engineering, gene therapy has become a potential approach in treating liver cancer. Suicide gene therapy, with its unique mechanisms, has been rapidly developed and attracted considerable attention [2, 3]. Using this approach, a suicide gene that encodes toxic protein under particular conditions can be delivered to target cells and effectively results in cell death, some suicide genes could also inhibit tumor cell growth by inducing apoptosis [4]. Thymidinekinase (TK) and E.colicytocinedeaminase (CD) are two most common suicide genes. Effective transfection and expression of TK/CD in tumor cells could facilitate both the direct killing effect and bystander effect of 5-FC/GCV [5]. In our previous study, we have constructed pEGFP-KDRP-CD/TK plasmid, which contains CD/TK double suicide gene driven by KDR promoter, and reported that the enhanced CD/TK gene transfection mediated by ultrasound microbubbles (MBs) was effective in killing breast cancer cells [6].

Successful gene therapy requires safe and efficient gene vectors and gene delivery methods. Current viral vector in gene therapy have several problems, including immunogenicity, potential tumorigenicity and low carrying capacity [7]. Liposomes as non-viral vectors can easily be swallowed by phagocytic system and cannot maintain a long life cycle [8]; therefore, it is crucial for researchers to develop more efficient and accurate gene vectors. Ultrasound-targeted microbubble destruction (UTMD) has been reported to provide a non-invasive, safe, and repeatable method for gene delivery [9–11]. Mechanical and cavitation effects generated by UTMD can enhance the temporary permeability of cell membrane and thus facilitate exogenous gene to enter into the targeted cells. The development of ultrasound contrast agent opens up a new idea of carrying target-delivery genes or drugs for chemotherapy in liver tumor patients. In previous study, by using ultrasound & high speed shearing method, we have constructed thrombus—targeted microbubbles and testified its physical and chemical properties [12]. In previous study, we have constructed thrombus-targeted microbubbles to stably target to blood clots (National invention patent, patent number: ZL200910305811.1).

**Fig 1:**
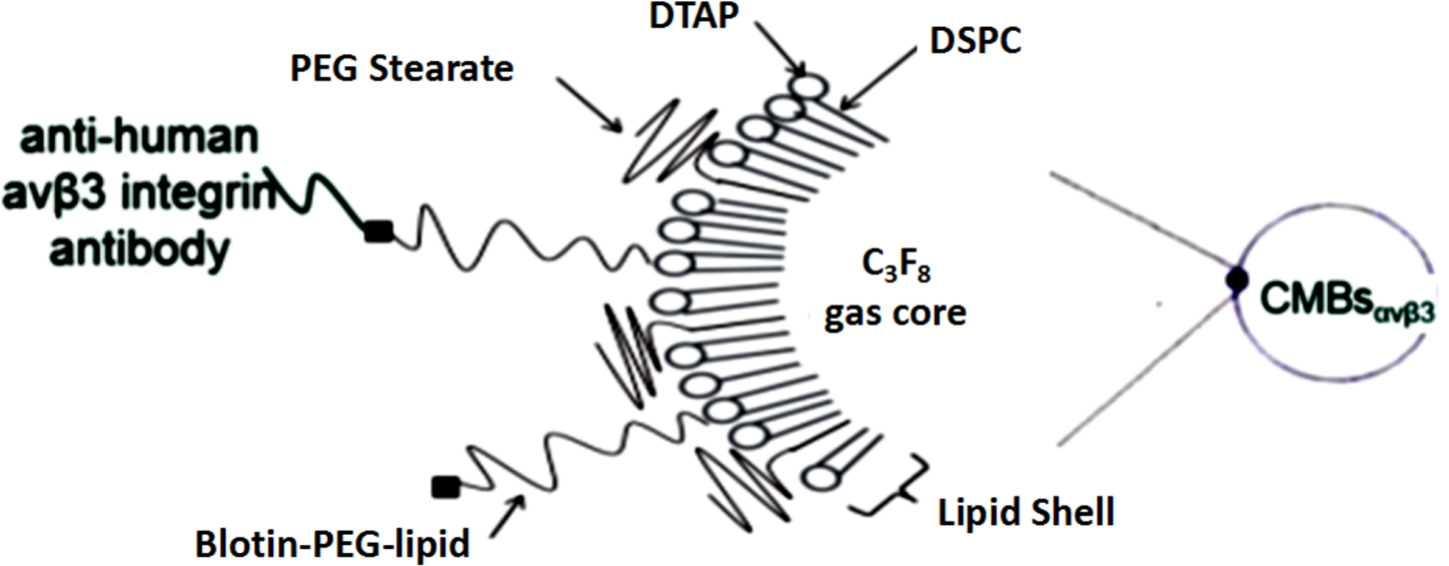
Schematic diagram of CMBs_αvβ3_ in specifically targeting human liver cancer HepG2 cells. The interaction between biotin and avidin is highly specific. Αvβ3 integrin antibodies on the surface of microbubbles can bind to α_v_β_3_ integrin receptors in HepG2 cells, which resulted in selective accumulation and longer resident time of CMBs_αvβ3_ in liver cancer tissue.

However, the transfection efficiency of these MBs is somewhat limited since some of the MB-carried plasmids do not accumulate in the target tissue. To further improve gene delivery efficiency, researchers have developed alternate approach by using modified microbubbles with a positively charged surface [13, 14]. CMBs can be combined with DNA through electrostatic interaction [15]. Moreover, plasmid DNA which charge-coupled onto the surface of CMBs are protected against endonuclease degradation [16]. To further improve plasmid transfection and gene delivery efficiency, our group has developed an inclusion of 1, 2-dis-tearoyl-3-trimethylammoniumpropane (Fig 1, DTAP; Avanti, Alabaster, AL, USA) imparted with a positive surface charge.

However, some studies report that the efficiency of UTMD-mediated gene delivery with CMBs is still limited [17]. Conjugating receptor ligands or antibodies to CMBs surface might be a promising approach to overcome this limitation. Angiogenesis is fundamental in tumor growth and metastasis [18,19]. Αvβ3 integrin, a well-established biomarker of tumor angiogenesis, is constitutively expressed in low level in quiescent endothelial cells but overexpressed in tumor endothelial cells during tumor angiogenesis [20, 21]. Because of its pivotal role in tumor growth and migration, α_v_β_3_ integrin has been selected as potential therapeutic target in cancers [22, 23]. As widely described in previous studies, microbubbles conjugated with designed antibody are able to specifically bind to target cells or tissues [24, 25].

In this study, to improve the tissue-specificity and transfection efficiency of gene therapy in liver cancer, we synthesized a cationic microbubble with an antibody against α_v_β_3_ integrin. Based on our previously demonstrated technique of ultrasound & high speed shearing in thrombus—targeted microbubbles, we constructed a CD/TK gene specific-delivery CMBs_αvβ3_ system in HepG2 cells (Fig 1). We showed that these CMBs_αvβ3_ could be accumulated around HepG2 cells and with increased DNA-binding capacity. Moreover, we found that, compared with non-targeted CMBs, CMBs_αvβ3_ would enhance 5-FC/TK-induced cell cycle arrest in S phase and facilitate apoptotic cell death in HepG2 cells.

## Materials and Methods

### Materials

1, 2-distearoyl-sn-glycero-3-phosphocholine (DSPC), dipalmitoyl phosphatidylcholine (DPPC) and biotinylated dipalmitoylphosphatidyl-ethanolamine (DSPE-PEG2000-Biotin) were purchased from Avanti PolarLipids Inc. (Alabaster, AL, USA). The inclusion of 1, 2-dis-tearoyl-3-trimethyl-ammonium-propane (DTAP; Avanti, Alabaster, AL, USA) imparted the agents with a positive surface charge [26].Human liver cancer HepG2 cells, human liver normal L-02 cells, SK-Herp-1 HCC cells, and lung adenocarcinoma A549 cells were purchased from Xiangya cell bank, Central South University (Changsha, China). Biotinylated anti-α_v_β_3_ antibody and rhodamine mouse anti-human immunoglobulin (Ig) G were obtained from Beijing Biosynthesis Biotechnology Co., LTD. Perfluorinated propane (C_3_F_8_) was purchased from the Special gas Co., LTD. Factory (Nanjing, China).

### Plasmids

As described in our previous study, the restructured plasmid pEGFP-KDRP-CD/TK coding for green fluorescent protein (GFP) contained CD/TK double suicide gene and was driven by KDR promoter [27]. The molecular weight of this plasmid is 2300 kDa with 3486bp. The plasmid was amplified and then isolated and purified using QIAGEN plasmid giga kit (Qiagen, Valencia, CA, USA) following the manufacturer’s protocol.

### Preparation of non-targeting cationic microbubbles (CMBs)

DSPC, DPPC, DTAP and DSPE-PEG-Biotin were mixed in a 5 ml plastic tube to form a suspension at a molar ratio of 46: 36: 8: 2. Following lyophilization, 1 ml of phosphate-buffered saline (PBS) was added to the samples to rehydrate them and then C_3_F_8_ gas was slowly injected into the container to replace the air. Samples were then agitated using an ultrasonic mechanical vibrator with high speed shearing method for 90 sec to form a milky white solution.

### Preparation of α_v_β_3_ antibody-conjugated cationic microbubbles (CMBs_αvβ3_)

Αvβ3 antibody was conjugated to the distal end of the DSPE-PEG2000-Biotin molecules through biotin-streptavidin coupling chemical method [28]. Briefly, 500μl CMBs (1x10^9^/ml) were mixed with 100 μg biotinylated anti-α_v_β_3_ antibody in an ultrasonic agitating reaction for 30 min. Then, after centrifugation at 50 g for 5 min, the upper layer (CMBs_αvβ3_) was washed with PBS three times and then collected. The morphology and particle distribution of CMBs_αvβ3_ was observed by optical microscopy. The particle size and surface potential were measured by a Zetasizer 3000HS (Malvern, Worcestershire, UK). All experiments were performed for five times.

### Determination of plasmid and α_v_β_3_ antibody binding onto the CMBs_αvβ3_ surface by fluorescent microscope and flow cytometry

To confirm conjugation of the αvβ3 antibody and plasmid to the CMBs, CMBs_αvβ3_ were diluted to a concentration of 7.8x10^7^ in 1.5ml sterile 0.9% saline, 500 μg plasmids and 500 mL of the CMBs_αvβ3_, and then mixed. The mixture was then incubated at room temperature with occasional shaking for 30 min. After incubation, unbound plasmids were removed by centrifugal washing for 5 min at 400g in 5 mL PBS for twice. The remaining pallet was resuspended in 0.5 mL PBS. To characterize these CMBs_αvβ3_, the plasmid was labeled with the nucleotide-avid fluorophore YOYO-1 and α_v_β_3_ ligand was labeled with 14.8ug/ml rhodamine mouse anti-human immunoglobulin (Ig) G. Fluorophore was incubated with the plasmid conjugated MB dispersion at 3 μg per 1 × 10^8^ MBs for 25 min at room temperature. And unbound fluorophores was removed by two rounds of centrifugal washing. These microbubbles were then counted and resuspended in PBS. Plasmid and the antibody conjugation to CMBs_αvβ3_ was observed under a fluorescence microscope (CKX41; Olympus, Tokyo, Japan). The binding rate was assessed by flow cytometry (Becton-Dickinson, Franklin Lakes, NJ).

### Rosette formation assay

HepG2 HCC cells and normal liver cell L-02 cells were cultivated in a 24-well plate with DMEM containing 10% fetal bovine serum in an incubator with 5% CO_2_ at 37°C. 5x10^4^/ml HepG2 cell and L-02 cells were digested with 2.5 g/L trypsin and incubated with CMBs_αvβ3_ or CMBs (1x10^8^/ml) at room temperature for 30 min. To block the rosette formation, HepG2 cells were pre-incubated with biotinylated α_v_β_3_ antibody at room temperature for 30 min before the cells were incubated with CMBs_αvβ3_ or CMBs for 30 min. After removing the unbound microbubbles, the resulting mixture (final volume 5 ml) was examined under microscope and the cells that formed rosette were counted.

### Gene transfection

100 μl HepG2 or L-02 cell suspension was mixed with 50 μl of CMBs_αvβ3_ containing pEGFP-KDRP-CD/TK plasmid. The ultrasonic therapy device ACUSON S2000 (SEMENS, USA) was preheated for 30 min. Then the ultrasound probe coated with coupling agent was put close to the bottom of the cell culture plates to sonicate the microbubbles (2 MHz, P = 0.75 W/cm2, t = 45s). 48 hours after sonication, EGFP expression was observed by an inverted fluorescence microscope. The transfection efficiency was determined using the formula: (Number of positive cells expressing EGFP/Total number of viewed cells) x100%.

### RT-PCR and Immunoblotting

The total RNA of the cells was extracted by Trizol Reagent. Upstream and downstream primers [7]of CD/TK was used for PCR reaction and 4 μl PCR amplified products were detected by electrophoresis on a 1.5% agarose gel. And CD/TK were measured by Western blot using anti-Thymidine kinase antibody and anti-CDA antibody (Milllipore, Billerica, MA).

Total cell lysates were harvested, subjected to 12% SDS-polyacrylamide gel electrophoresis and then transferred to polyvinylidene difluoride membranes (Roche, Basel, Switzerland). Immunoblotting involved incubation with the primary antibodies followed by the addition of secondary antibodies conjugated to horseradish peroxidase (Cell Signaling) to facilitate detection. Subsequently, enhanced chemiluminescence reagent (Cell Signaling) was added to develop the blots.

### Analysis of cell cycle arrest and apoptosis

To determine the effects of CMBs_αvβ3_ containing pEGFP-KDRP-CD/TK plasmid on the cell cycle, propidium iodide (PI) staining after 75% alcohol fixation was used, followed by flow cytometry analysis. Featured apoptotic cell death was detected by optical microscope. 5x10^4^/ml HepG2 cells with or without treatment were observed at 24, 48 and 72 hours after treatment. For apoptotic cells, DNA fragmentation was detected by the terminal deoxynucleotidyltransferase mediated dUTP nick end labeling (TUNEL) assay (Roche Applied Science, Pennsburg, Germany). For the TUNEL assay, cells with or without treatment were cultures were fixed with 4% paraformaldehyde in PBS for 20 min, and then washed with PBS for 30 min and incubated with permeabilization solution (0.1% Triton X-100 in 0.1% sodium citrate) for 2 min on ice. Cells were washed twice and incubated in a humidified atmosphere with TUNEL reaction mixture for 60 min at 37°C in the dark. Then, 50ul POD was added and incubated for 20–30 min at 37°C. After washed by PBS for three times, 50-100ul DAB was added and incubated for 5–10 min. After washed by PBS, hematoxylin stained for 10–30 sec and then was washed away. Finally, cells were analyzed under an optical microscope.

### MTT assay

After treatment, 50 μl MTT was added into each well in 96-well plates, and then mixed with cells. And then the cells were treated with different concentration of 5-FC (20mg/l,40mg/l,60mg/l….) and GCV (0.02 mg/l,o.1mg/l,0.25mg/l….) according to the different groups. After incubation with MTT for 30 minutes, cell viability was measured by the optical density (OD) value of each well by an enzyme-linked immunosorbent assay instrument at 490 nm. Each concentrationand treatment time point has three duplicates. The rate of cellular inhibition was determined using the formula: (1-OD value in treatment group/OD value in control group) x100%.

### In vivo treatment

Tumor xenograft animal model was constructed following protocol [29].30 nude mice bearing HepG2 liver cancer were randomly divided into 5 groups. For group 1, normal saline (NS) were administrated by intraperitoneal injection daily. In group 2, 160mg/kg5-FC and 100mg/kg GCV were administrated by intraperitoneal injectiondaily. In group 3,40ug plasmid was injected from posterior venous plexus on day 1 and 2, with 5-FC + GCV daily injection. In group 4, 80ul microbubble was injected from posterior venous plexus and followed by ultrasound wave irradiation (1MHz, 2w/cm^2^, 50% duty cycle, irradiation for 10s with 10s stop, 5 min in total) for 2 days. In group 5,2 day treatment of 40ug plasmid + 80ul microbubbleand ultrasound wave irradiation was coupled with 5-FC + GCV daily. The total treatment duration was 10 days.The tumor growth was observed and tumor volumes were calculated. Then, HepG2 xenografts were removed and weighted. Immunohistochemical staining was used to detect the expression levels of CD31. Apoptotic cells were detected by TUNEL. The detailed procedures were described in reference paper [30].

### Statistical analysis

All data are expressed as mean ± standard deviation (SD), unless otherwise noted. Differences among groups were analyzed by one-way ANOVA and LSD method for multiple comparisons among groups using the SPSS software package (Version 19.0 for windows, SPSS, Chicago, Illinois, USA) with p < 0.05 considered statistically significant.

## Results

### Morphology and coupling assessment of microbubble

**Fig 2:**
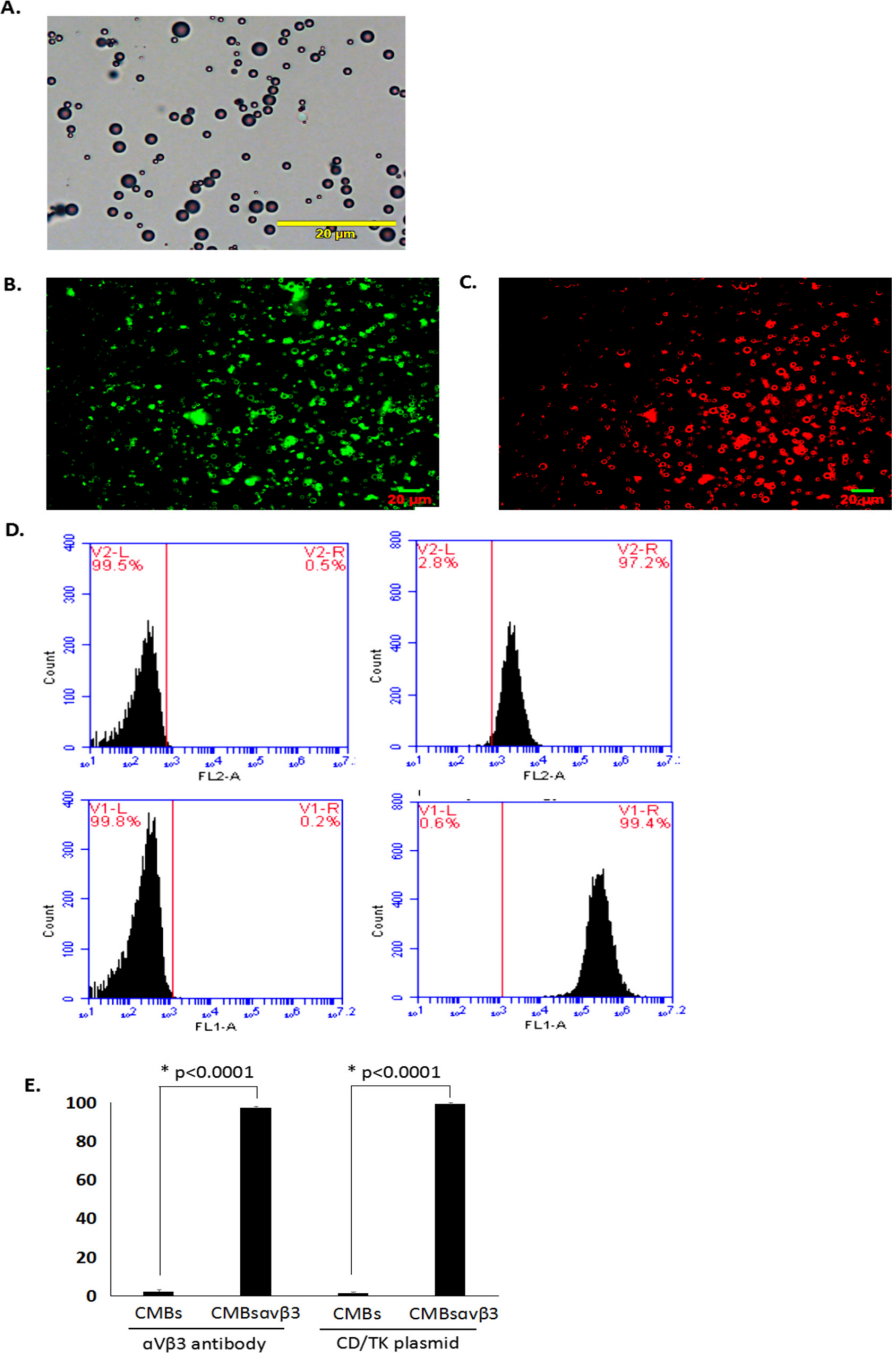
Morphology and fluorescence image of CMBs_αvβ3._ A. Under light microscopy (magnification, x400), microbubbles were uniformly distributed with no visible aggregation. Each photo is representative of three experiments. B. Green fluorescence was observed on the surface of CMBs_αvβ3_ using immunofluorescent microscopy. Each photo is representative of three experiments. C. Red fluorescence was observed on the surface of CMBs_αvβ3_ using immunofluorescent microscopy. Each photo is representative of three experiments. D. Flow cytometry analysis of the α_v_β_3_ antibody and plasmid combined to CMBs_αvβ3_ or CMBs. Each histogram is representative of three experiments. E. Comparison of the α_v_β_3_ antibody and plasmid binding efficiencies in cells treated with CMBs_αvβ3_ or cells treated with CMBs (mean ± SD of three experiments; *p* < 0.0001).

The morphology of polydisperse gas-encapsulated CMBs_αvβ3_ was assessed by light microscopy. As shown in Fig 2A, CMBs_αvβ3_ was homogeneously distributed and relatively uniform without adhesion and aggregation. As measured by electrozone sensing with a Coulter counter, the mean diameter of CMBs_αvβ3_ was 1.97 μm, and the concentration was 5.2x10^5^ bubbles/mL.

To assess the conjugation strength of plasmid and α_v_β_3_ integrin antibody with CMBs_αvβ3,_ spectrophotometer and flow cytometry assays were adopted. After incubation with CMBs_αvβ3_, plasmid and the antibody conjugation to CMBs_αvβ3_ was observed under fluorescence microscope. The green and red fluorescence signal indicated the binding strength of YOYO-1–labeled plasmid and Rhodamine–labeled α_v_β_3_ integrin antibody to CMBs_αvβ3_, respectively. As shown in Fig 2B, bright green fluorescence signals were observed on the surface of CMBs_αvβ3_ and demonstrated that the plasmid was tightly connected to the CMBs_αvβ3_. The red fluorescent signals in Fig 2C suggested a successful conjugation of that α_v_β_3_ integrin antibody with CMBs_αvβ3_.

### Combination of the antibody and plasmid to CMBs_αvβ3_

Both the plasmid and α_v_β_3_ integrin antibody were conjugated on the surface of cationic microbubbles. The binding efficiency of plasmid and α_v_β_3_ integrin antibody with CMBs_αvβ3_ was analyzed by flow cytometry. As shown in Fig 2D and 2E, compared with CMBs, about 97.2% CMBs_αvβ3_ were combined with the α_v_β_3_ antibody (Fig 2D, right-up panel) while about 99.4% CMBs_αvβ3_ combined with the plasmid (Fig 2D, right-bottom panel). These findings demonstrated a tight and efficient combination of the antibody and plasmid to CMBs_αvβ3_.

### Targeting efficiency of CMBs_αvβ3_ in Hep2 and L-02 cells

**Fig 3:**
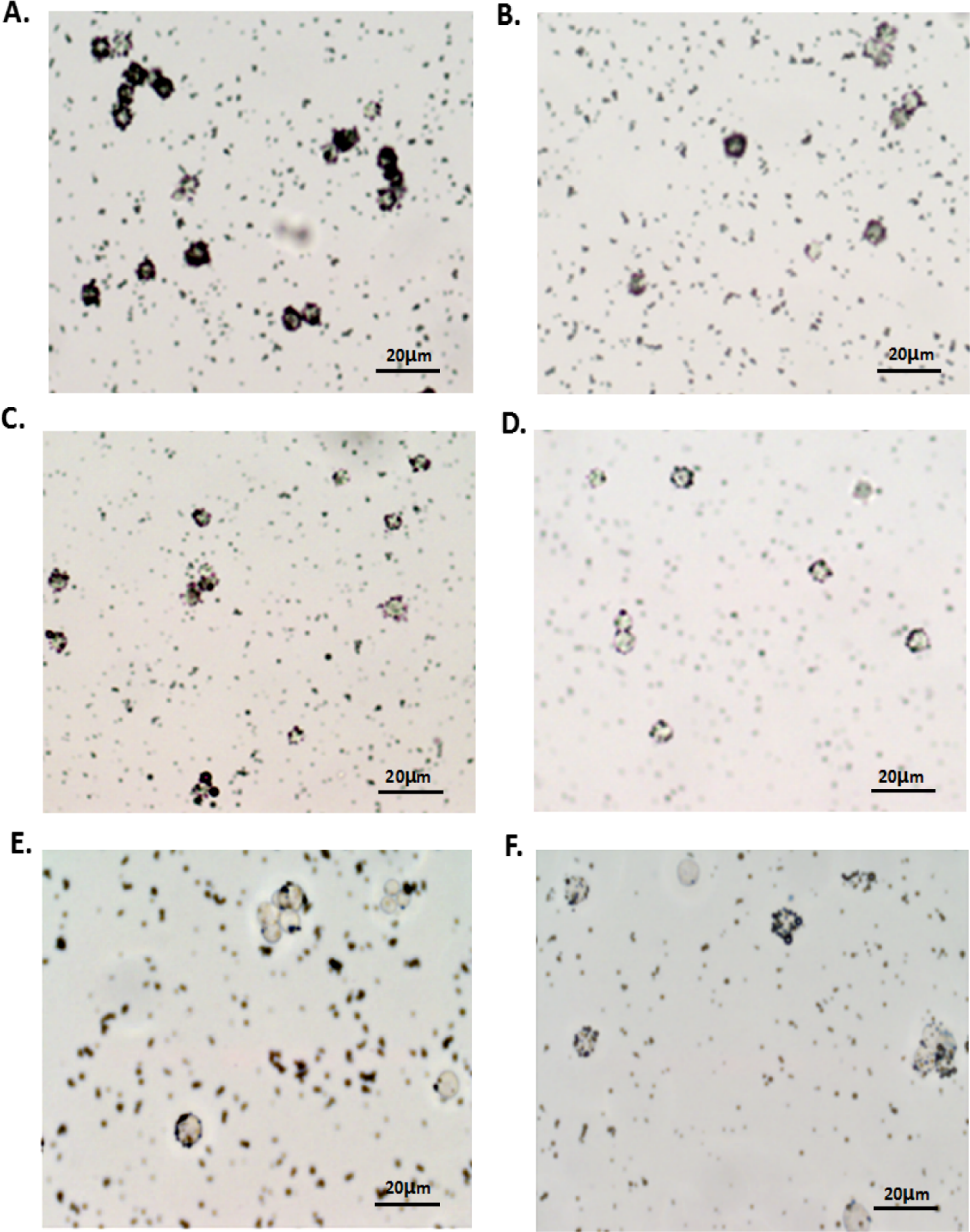
Target-searching image in vitro (400×). (A, C) Light microscope image of specific binding in the targeted group; (B, D) light microscope image of binding in the non-targeted group. (E, F) light microscope image of binding after using anti-α_v_β_3_ monoclonal antibody. Each photo is representative of three experiments.

To examine the targeting efficiency of to HepG2 cells, we compared the rosette formation rate (the number of CMBs_αvβ3_ positive cells or CMBs positive cells in 100 cells) in HepG2 cells and L-02 cells (Table 1). Cells combined with more than four CMBs_αvβ3_ or CMBs was considered as CMBs_αvβ3_ or CMBs positive cells. As observed under the light microscope (Fig 3), when HepG2 cells and normal hepatocytes L-02 cells were incubated with CMBs_αvβ3_, CMBs_αvβ3_ aggregated around HepG2 and L-02 cells and formed a rosette-like structure. However, pre-incubation with α_V_β_3_ antibody could block this rosette formation. The rosette formation rate in HepG2 cells (Fig 3A) and L-02 cells (Fig 3B) were 52% and 35%, respectively, indicating a significant higher targeting efficiency of CMBs_αvβ3_ to HepG2 cells than to L-02 cells (p<0.05). The rosette formation rates of CMBs in HepG2 cells (Fig 3C) and L-02 cells (Fig 3D) were 13.33 ± 6.33% and 8.33 ± 1.52%, respectively, much lower than the rosette formation rates of CMBs_αvβ3_, indicating a significantly greater adhesion of CMBs_αvβ3_ to HepG2 cells than CMBs. In addition, after pretreated with different concentrations of anti- α_v_β_3_ monoclonal antibody, the rosettes formation rates declined significantly (p<0.05), and little CMBs and CMBs_αvβ3_ were aggregated around HepG2 cells(Fig 3E and 3F).

**Table 1:** Comparison of the rosette formation rates in each group (n = 6, % mean ± SD of three experiments).

Group	L-02 cells	HepG2 cells	
		Without pre-incubation	Pre-incubation with α_v_β_3_ antibody
CMBs_αvβ3_	34.67 ± 2.08	51.67 ± 5.69^a^^,^^b^	3.67 ± 0.57^b^
CMBs	8.33 ± 1.52^c^	13.33 ± 6.33^a^^,^^c^	7.33 ± 1.52^c^

Note

a. In HepG2 cells without pre-incubation, the difference of rosette formation rates in CMBs_αvβ3_ group and CMBs group was statistically significant, *p* < 0.05.

b. In HepG2 cells with CMBs_αvβ3_, the difference of rosette formation rates between group with α_V_β_3_ antibody pre-incubation and group without pre-incubation was statistically significant, *p* < 0.05.

c. The difference of rosette formation rates of CMBs in L-02 cells, HepG2 cells without any pre-incubation, and HepG2 cells with α_V_β_3_ antibody pre-incubation was not statistically significant, *p* > 0.05.

### Transfection efficiency of CMBs_αvβ3_ in HepG2 and L-02 cells

**Fig 4:**
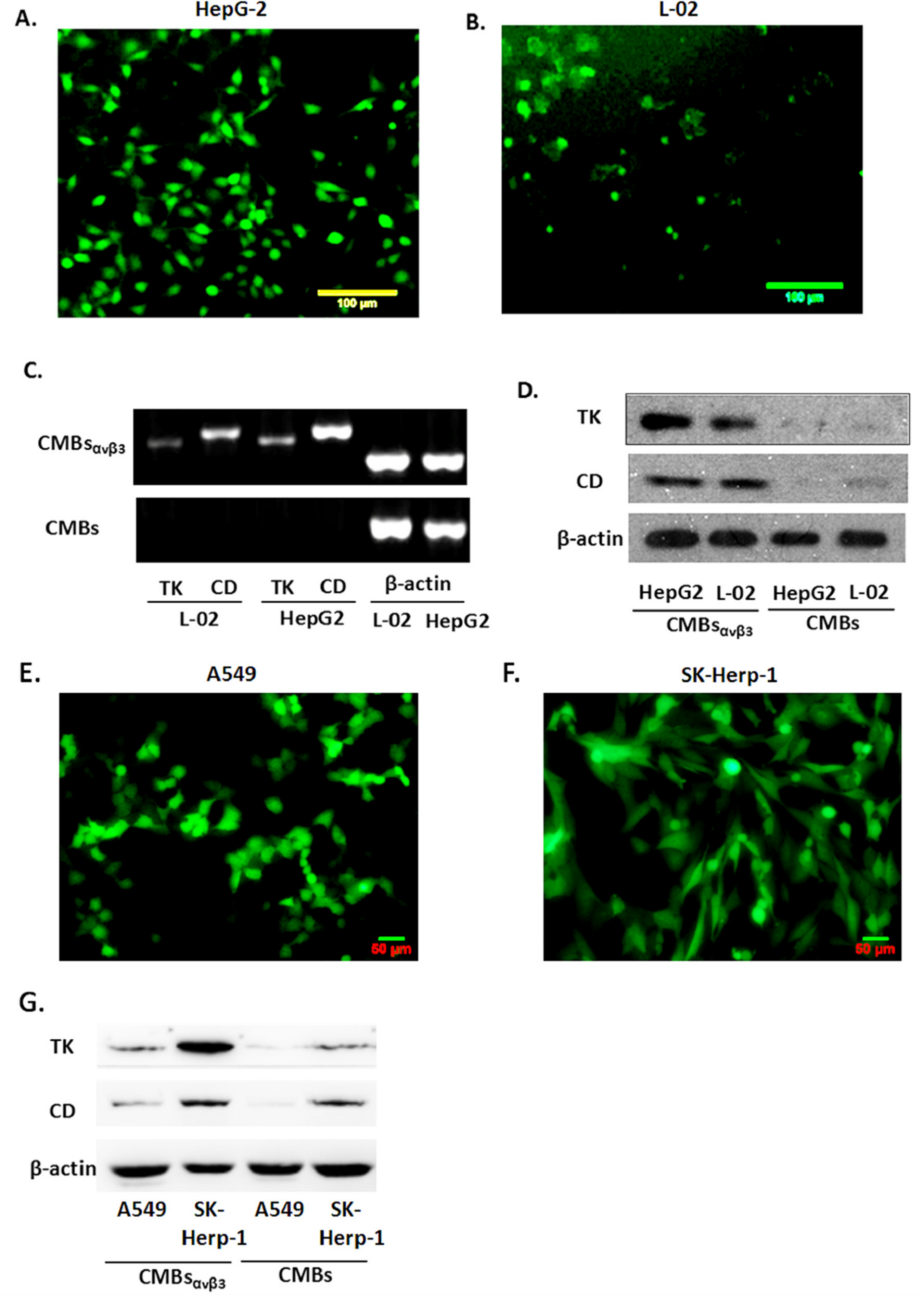
Transfection efficiency of CMBs_αvβ3_ and Expression of CD/TK in HepG2 and L-02 cells. A and B. EGFP expression levels in HepG2 cells (A) and L-02 cells (B) were detected using fluorescence. 10 visions were chosen at random and each experiment was repeated for three times (mean ± SD of three experiments). C. mRNA expression of the target gene CD/TK in HepG2 and L-02 cells was evaluated by RT-PCR. Bands at 381bp (CD) and 355bp (TK) were observed in all groups of both HepG2 and L-02 cells. Each photo is representative of three experiments. D. Protein expression level of CD/TK in HepG2 and L-02 cells. Lane1:L02; Lane2:HepG2; Lane3: L02+CD/TK; Lane4:HepG2+CD/TK. Each photo is representative of three experiments. E and F. EGFP expression levels in A549 cells (E) and SK-Herp-1 cells (F) were detected using fluorescence. 10 visions were chosen at random and each experiment was repeated for three times (mean ± SD of three experiments). G. Protein expression level of CD/TK in A549 and Ssk-Herp-1 cells. Each photo is representative of three experiments.

As demonstrated in Fig 4, at 24 h after CMBs_αvβ3_ transfection, EGFP expression levels in HepG2 cells and L-02 cells were evaluated by fluorescence microscope (Fig 4A and 4B).The number of cells expressed EGFP was counted (200×). The transfection efficiency was determined using the formula: (100×Number of positive cells expressing EGFP/Total number of viewed cells) %. The percentage of positive cells were 81.67 ± 3.51% in HepG2 cells and 37.67 ± 1.53% in L-02 cells (P < 0.05), suggesting a significant higher transfection efficiency of CMBs_αvβ3_ in HepG2 cells than in L-02 cells.Besides HepG2 cells, CMBs_αvβ3_ transfection was detected in lung adenocarcinoma A549 cells and SK-Herp-1 HCC cells (Fig 4E and 4F).

### Expression of CD/TK in HepG2 and L-02 cells

After pEGFP-KDRP-CD/TK was transfected into HepG2 and L-02 cells, we found that there was a marked mRNA expression of the target gene CD/TK in HepG2 and L-02 cells by RT-PCR detection (Fig 4C). Bands at 381bp (CD) and 355bp (TK) were observed in both HepG2 and L-02 cells with CMBs_αvβ3_ CD/TK plasmid transfection. CD/TK bands were not observed in HepG2 and L-02 cells with only CMBs treatment. Moreover, Fig 4C showed that compared to L-02 cells, CD/TK mRNA expression was significantly higher in HepG2 cells with CMBs_αvβ3_ CD/TK plasmid transfection. Immunoblotting results were shown in Fig 4D. CD/TK protein was expressed in both L-02 and HepG2 cells with CMBs_αvβ3_ CD/TK plasmid transfection. The highest TK expression level was observed in HepG2 cells with CMBs_αvβ3_ CD/TK plasmid transfection, which was consistent with the RT-PCR results. Moreover, CD/TK protein expression level was higher in SK-Herp-1 HCC cells than A549 cells (Fig 4G).

### CMBs_αvβ3_ facilitates 5-FC/GCV-induced cell cycle arrest at S phase in HepG2 cells

**Fig 5:**
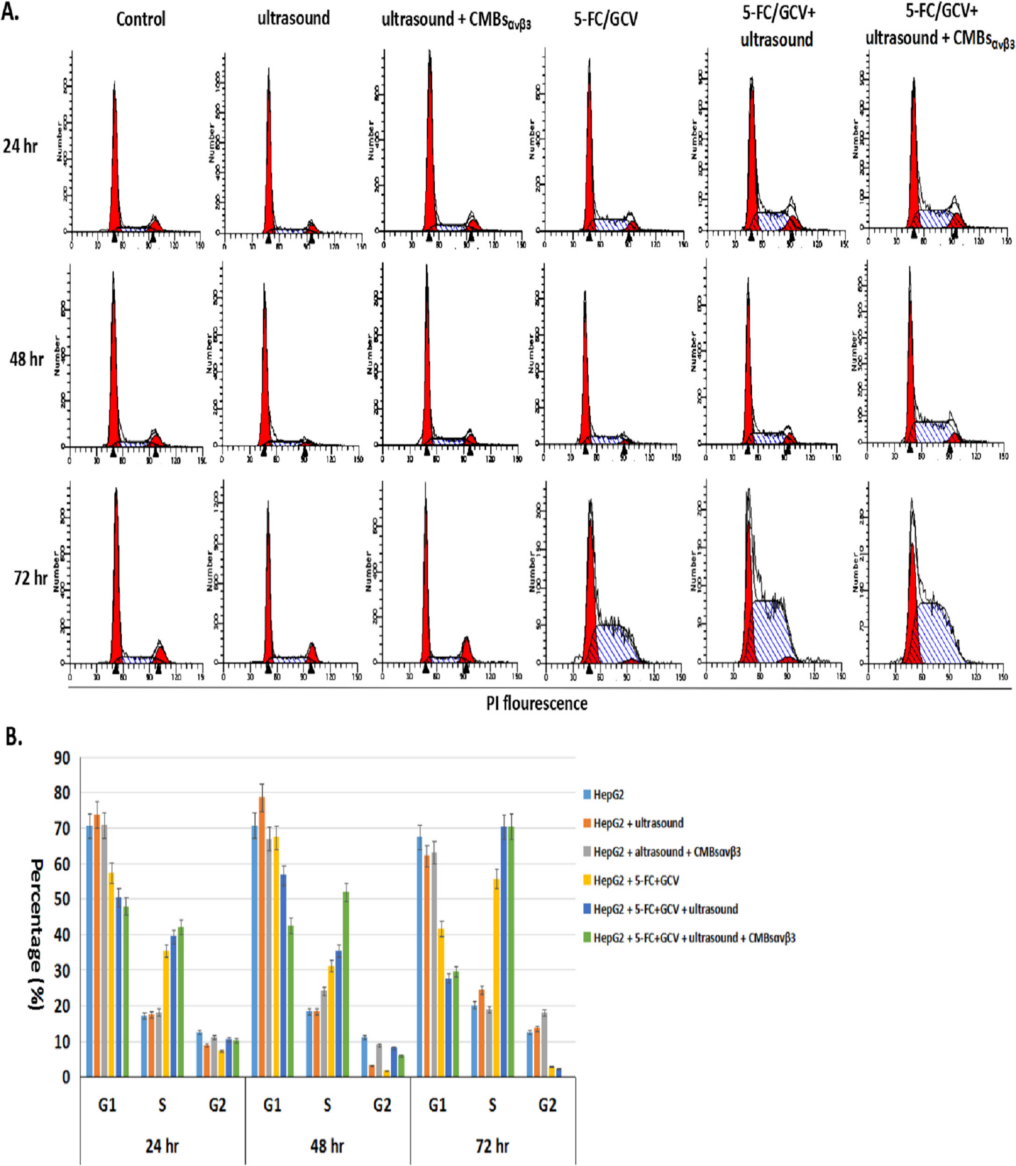
Effect of CMBs_αvβ3_ in 5-FC / GCV—induced cell cycle arrest in HepG2 cells. A. Cell cycle analysis of untreated HepG2 cells and HepG2 cells treated with 5-FC / GCV, 5-FC / GCV + ultrasound, or 5-FC / GCV + ultrasound + CMBs_αvβ3_for 24, 48 and 72 hours, as analyzed by flow cytometry and PI staining. Each histogram is representative of three experiments. B. The effects of 5-FC / GCV + ultrasound + CMBs_αvβ3_treatment on the cell cycle distribution of HepG2 cells were analyzed using Multicycle software program (Phoenix Flow System) (mean ± SD of three experiments; *p < 0.01).

To investigate the effect of CMBs_αvβ3_ in 5-FC/GCV-treated HepG2 cells, PI staining and flow cytometry were firstly adopted to analyze the cell cycle distribution. As shown in Fig 5, compared with untreated HepG2 cells, 5-FC (64ug/ml) /GCV (320ug/ml) could arrest 35.3%, 31.05% and 55.68% HepG2 cells at S phase of the cell cycle at 24, 48 and 72 hours after treatment, respectively. When combined with ultrasound and CMBs_αvβ3_, 41.94%, 51.81% and 70.40% HepG2 cells were arrested in S phase of the cell cycle at 24, 48 and 72 hours after treatment, respectively, which were significantly higher than 5-FC (64ug/ml) / GCV (320ug/ml) treatment alone. These findings implied that CMBs_αvβ3_ could facilitate 5-FC/GCV-induced cell cycle arrest at S phase in HepG2 cells.

### CMBs_αvβ3_ induces apoptosis in HepG2 cells

**Fig 6:**
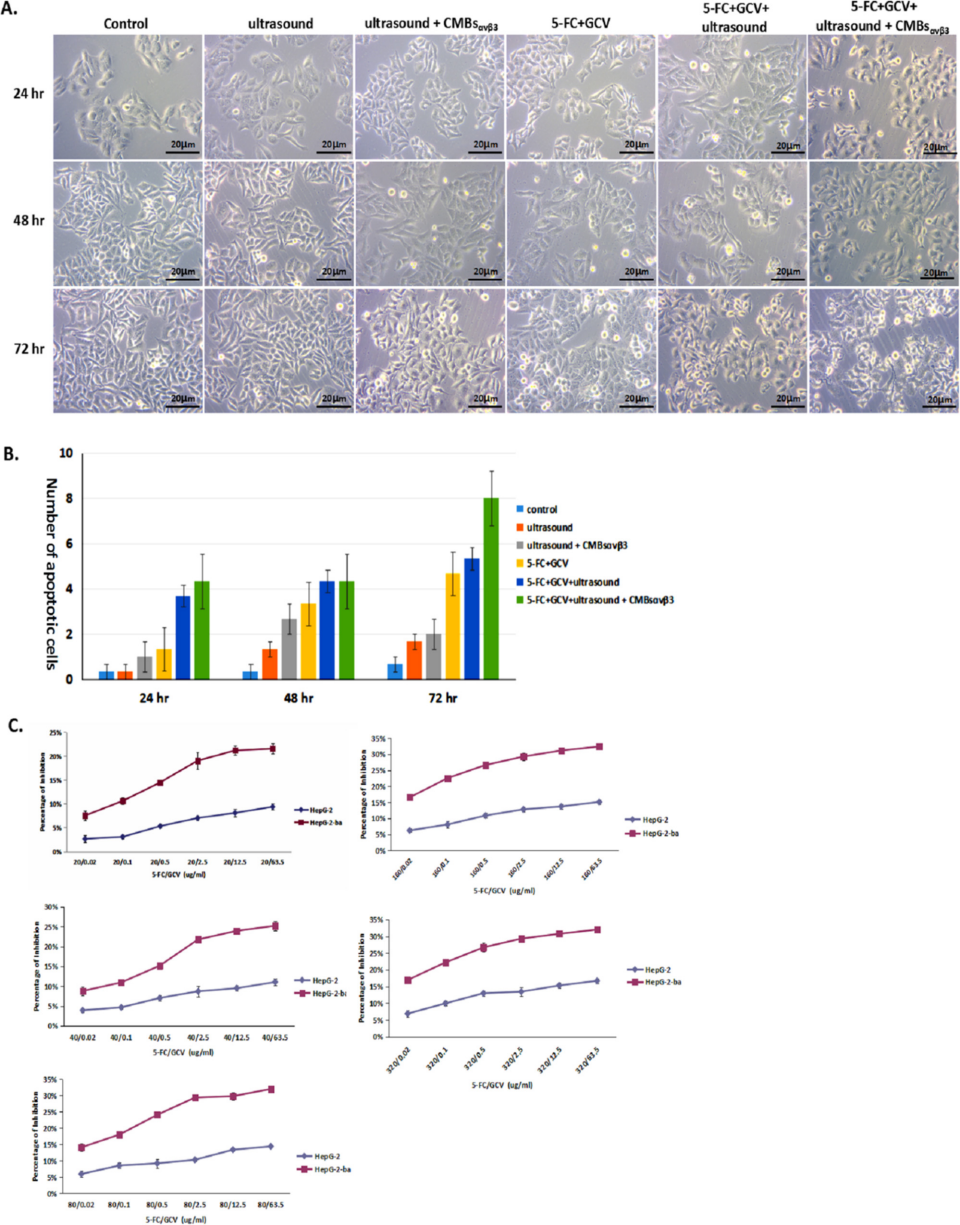
Effect of CMBs_αvβ3_in 5-FC / GCV—induced anti-proliferation in HepG2 cells. A. HepG2 cells were treated with ultrasound alone, ultrasound + CMBs_αvβ3_,5-FC / GCV, 5-FC / GCV + ultrasound, or 5-FC / GCV + ultrasound + CMBs_αvβ3_for 24, 48 and 72 hours. Untreated HepG2 cells were served as control. Featured apoptotic cell death was observed under optical microscope (Magnitude×40). B. Numbers of apoptotic cells in each treatment group were counted for five different visual fields (mean ± SD of three experiments; *p < 0.05). C. The effect of CMBs_αvβ3_in 5-FC / GCV—induced anti-proliferation in HepG2 cells was measured by MTT assay. 5-FC / GCV with ultrasound plus CMBs was served as control (mean ± SD of three experiments; *p < 0.05).

As shown in Fig 6, no obvious morphological change was occurred in untreated HepG2 cells (control) within 72 hours, while some of HepG2 cells underwent apoptotic cell death with characteristic morphological changes at 48 and 72 hours after 5-FC (64ug/ml) /GCV (320ug/ml) treatment.Moreover, when5-FC/GCV combined with ultrasound and CMBs_αvβ3_, more apoptotic HepG2 cells were observed at 24, 48 or 72 hours after treatment, which were significantly higher than 5-FC / GCV treatment alone.MTT assay demonstrated that the inhibition rate of HepG2 cells treated with 5-FC / GCV and CMBs_αvβ3_ was significantly greater than that of cells treatedwith only 5-FC+GCV at different concentrations(Fig 6C). Also, in SK-Herp-1 HCC cells, 5-FC / GCV and CMBs_αvβ3_was more effective than 5-FC+GCV alone (S1 Fig).

**Fig 7:**
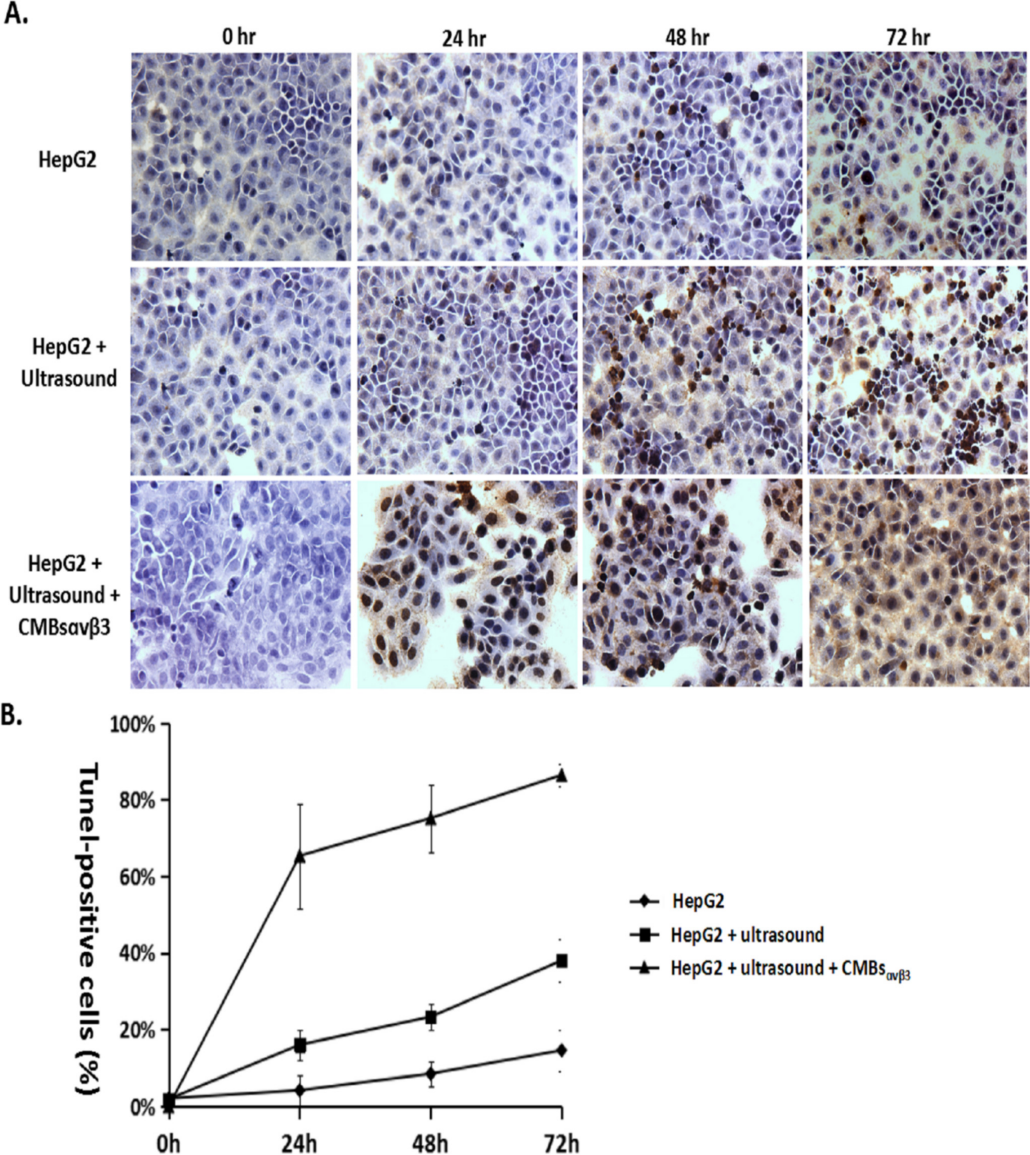
A. HepG2 cells were treated with5-FC / GCV, 5-FC / GCV + ultrasound, or 5-FC / GCV + ultrasound + CMBs_αvβ3_for 24, 48 and 72 hours. TUNEL-positive apoptotic cells were observed in HepG2 cells (Magnitude×40). B. Numbers of TUNEL-positive cells in each treatment group were counted for five different visual fields (mean ± SD of three experiments; *p < 0.05).

To further confirm 5-FC / GCV + CMBs_αvβ3_—induces apoptosis in HepG2 cells, TUNEL assay was adopted. Fig 7A illustrated HepG2 cells with TUNEL staining after UTMD-mediated CD/TK plasmid delivery. Nuclei were stained blue with DAPI. In 5-FC/GCV-treated HEPG2 cells (control), the TUNEL-positive (apoptotic cells) were minimal at 24 and 48 hours. However, in HepG2 cells treated with 5-FC/GCV and ultrasound plus CMBs_αvβ3_, the number of TUNEL-positive apoptotic cells increased significantly at 24 and 48 hours after treatment (p<0.05). Quantitative analysis demonstrated that the number of apoptotic cells (TUNEL-positive cells) was highest in HepG2 cells treated with 5-FC/GCV and ultrasound plus CMBs_αvβ3_ (p<0.05).

### CMBs_αvβ3_ suppresses tumor growth in HepG2 xenograft mice

**Fig 8:**
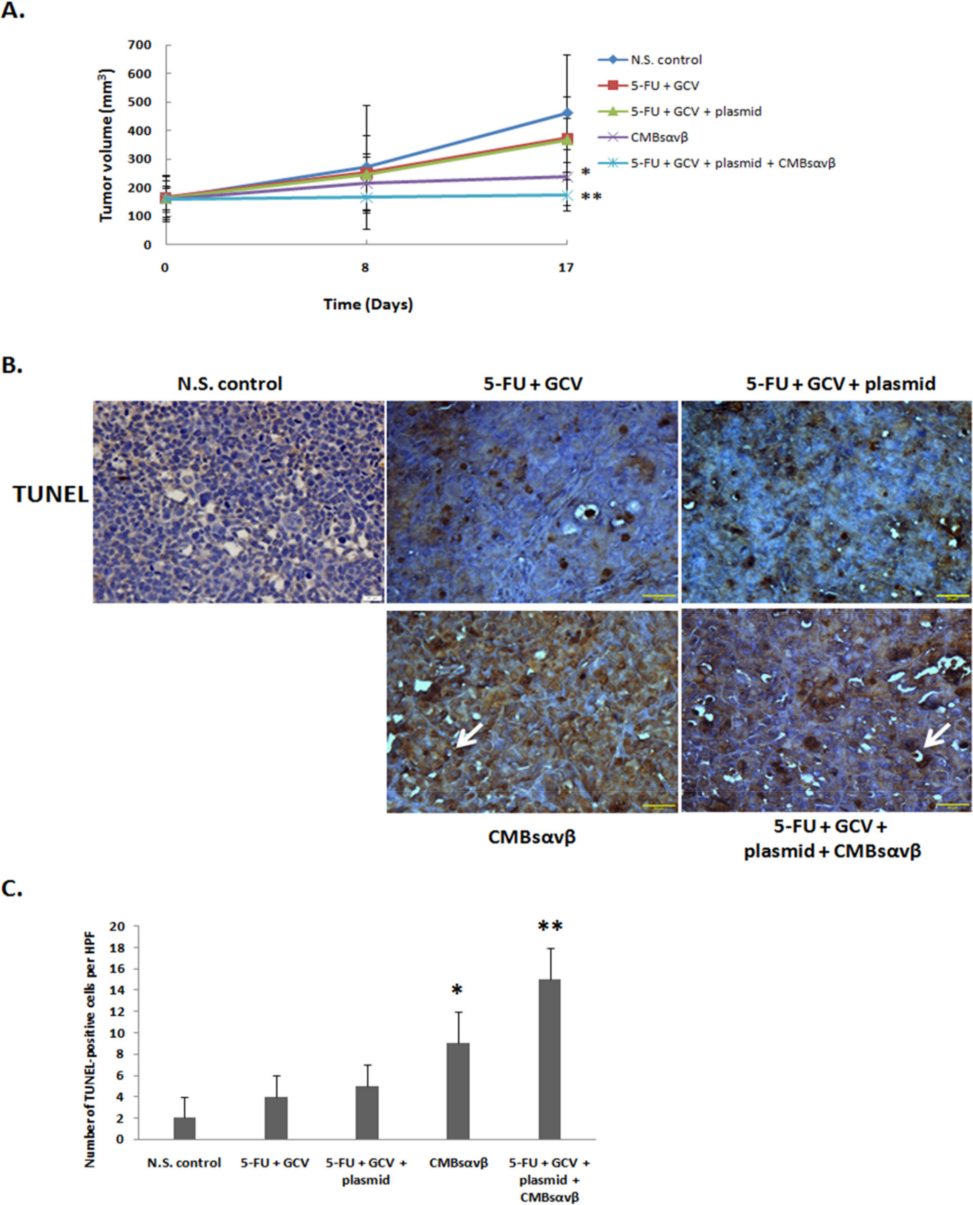
A. HepG2-bearing nude mice were treated with NS, 5-FC / GCV, 5-FC / GCV + plasmid, CMBs_αvβ3_or 5-FC / GCV + plasmid + CMBs_αvβ3_.Tumor volume were measured on day 0, 8 and 17. On day 17, the tumor volume in CMBs_αvβ3_ group were significantly lower compared with NS (* p<0.05). The tumor volume in 5-FC / GCV + plasmid + CMBs_αvβ3_ group were significantly lower compared with NS, 5-FC / GCV, 5-FC / GCV + plasmid group (**P<0.05). B. TUNEL-positive apoptotic cells were observed in HepG2 tumor section (Magnitude×40). C. Numbers of TUNEL-positive cells in each treatment group were counted for five different visual fields (mean ± SD of three experiments). CMBs_αvβ3_treatment induced more TUNEL-positive cells thanNS treatment (*p < 0.05). The number of TUNEL-positive cells was significantly higher in 5-FC / GCV + plasmid + CMBs_αvβ3_treatmentgroup than NS, 5-FC / GCV or 5-FC / GCV + plasmid group (**p<0.05).

To investigate the anti-tumor effect of CMBs_αvβ3_ in vivo, we applied HepG2 xenograft mice model. As shown in Fig 8A, compared with control group (NS), 5-FU +GCV or 5-FU + GCV + plasmid could not significantly suppress tumor volume at seven days after treatment (treatment duration was 10 days). However, when5-FU + GCV combined with plasmid + CMBs_αvβ3_ was able to effectively suppress tumor growth, compared with 5-FU + GCV or 5-FU + GCV + plasmid treatment (p<0.05). Moreover, compared with control group, both CMBs_αvβ3_treatment alone and 5-FU + GCV combined with plasmid + CMBs_αvβ3_were able to significantly suppress tumor volumes (p<0.05).

To determine whether CMBs_αvβ3_treatment facilitate 5-FU + GCV–induced apoptotic cell death in liver cancer cells in vivo, tumor sections from HepG2-bearing nude mice were stained with TUNEL to identify the apoptotic cell population. As shown in Fig 8B and 8C, treatment with5-FU + GCV or 5-FU + GCV + plasmid did not appreciably induce apoptosis. Treatment with CMBs_αvβ3_ alone and 5-FU + GCV combined with plasmid + CMBs_αvβ3_ stimulated a substantially increased number of TUNEL-positive cells in tumor sections, as indicated by vacuolated and brown-nuclear cells (arrow heads). 5-FU + GCV with plasmid + CMBs_αvβ3_ induced more TUNEL-positive cells than 5-FU + GCV alone (p<0.05, Fisher’s exact test).

## Discussion

CMBs_αvβ3_ were successfully synthesized and conjugated with a α_v_β_3_ antibody using bio-conjugation. CMBs_αvβ3_ had a regular shape and good dispersion, and showed significantly greater adhesion to HepG2 cells than CMBs, specifically when targeting HepG2 cells. Importantly, our data also demonstrated that delivery of the pEGFP-KDRP-CD/TK plasmid by CMBs_αvβ3_ resulted in a substantially greater gene transfection efficiency. Therefore, this CMBs_αvβ3_ is an important advance that could be translated clinically to improve gene delivery in liver cancer therapy.

We previously established a CD/TK suicide gene system in which CD/TK gene expression was modified by tumor tissue-specific promoter KDRP, as pEGFP-KDRP-CD/TK. The transfection of this CD/TK suicide gene system was mediated by ultrasound microbubbles and led to specific killing-effect on cancer cells[6]. Though UTMD has been reported to provide a non-invasive, safe, and repeatable method for gene delivery[10, 11, 31] and contrast agents intravenously injected are used as carriers for genes or drugs, the therapeutic effects were somewhat limited because the plasmids did not accumulate in the target tissue. Ways to improvement include enhancing the genes carrying by the microbubble contrast agents and increasing the targeting abilities of the microbubble through changing its surface feature.

Positively charged microbubbles possess increased carrying capacity for negatively charged plasmid DNA[14, 15]. However, some studies still report the limited efficiency of UTMD-mediated gene delivery even with CMBs[17]. To increase the therapeutic potential of CMBs, it’s a hopeful approach by conjugating receptor ligands or antibodies to their surface. Angiogenesis, the growth of new blood vessels from existing vessels, is a fundamental component of tumor growth and metastasis [18, 19]. α_v_β_3_ integrin is the best-characterized molecular markers of tumor angiogenesis and plays a pivotal role in tumor growth and migration, thus it has been selected as targets for therapeutic strategies successfully in cancer [20, 21]. Therefore, microbubbles conjugated with antibodies against α_v_β_3_-integrin would share feasible targeting ability to tumor tissues.

The value of our CMBs_αvβ3_ is its capacity for targeted tissue binding and efficient gene delivery. Here we synthesized a CD/TK delivery cationic microbubble as CD-TK/ CMBs_αvβ3_ conjugated with α_V_β_3_ antibody using poly (ethylene glycol)-biotin–streptavidin. In this study, firstly, the fluorescence of GFP or anti-α_V_β_3_ approved the CD/TK gene and α_V_β_3_ have been successfully carried by the microbubble, with further confirmed through flow cytometry. Then we demonstrated that the CMBs_αvβ3_ exhibited significantly greater human liver HepG2 cell binding compared with the non-targeted CMBs. And the adhesion of CMBs_αvβ3_ to human liver HepG2 cell and L-02 cells were reduced significantly after pre-administration of anti-α_V_β_3_ monoclonal antibody, which indicates the adhesion is liver tumor cell specific and α_V_β_3_ mediated. Importantly, our data also demonstrated that CMBs_αvβ3_ delivery of the CD/TK plasmid resulted in substantially great gene transfection efficiency, validated by fluorescence, PCR and western blot. In this study, we also found that CMBs_αvβ3_ could facilitate 5-FC/GCV-induced cell cycle arrest at S phase in HepG2 cells after PI staining and flow cytometry. And MTT assay demonstrated that the inhibition rate of HepG2 cells treatedwith 5-FC / GCV and CMBs_αvβ3_ was significantly greater than that of cells treated with only 5-FC+GCV at different concentrations. Quantitative analysis demonstrated that the number of apoptotic cells (TUNEL-positive cells) was highest in HepG2 cells treated with 5-FC/GCV and ultrasound plus CMBs_αvβ3_ (*p*<0.05).Therefore this CMBs_αvβ3_-based CD/TK suicide gene delivery system is definitely effective and an important advance that could be translated clinically to improve suicide gene therapy for liver cancer and other malignant tumors, though in vivo experimental research are warranted before its clinical translation.

## Conclusions

CMBs_αvβ3_ specifically targeting human liver cancer HepG2 cells were successfully prepared through biotin-avidin mediation. Numerous problems of targeting contrast agents remain, including the stability in the blood or the induced immune response, which require further investigation. However, our study demonstrated that the novel CMBs_αvβ3_ were able to specially target the CD/TK gene to the HepG2 cells and could be potentially applied in targeted gene delivery and therapy of cancer. This technique may be of significant utility in the field of molecular imaging, both in the setting of research and clinical practice.

## Supporting Information

S1 Fig The effect of CMBs_αvβ3_ in 5-FC / GCV—induced anti-proliferation in SK-Herp-1 HCC cells was measured by MTT assay. 5-FC / GCV with ultrasound plus CMBs was served as control (mean ± SD of three experiments; *p < 0.05).
(JPG)

S2 Fig The effect of CMBsαvβ3 in 5-FC / GCV—induced anti-proliferation in A549 cells was measured by MTT assay. 5-FC / GCV with ultrasound plus CMBs was served as control (mean ± SD of three experiments; *p < 0.05).
(JPG)
