# Supplementary figures and images for: Effects of Cationic Microbubble Carrying CD/TK Double Suicide Gene and αVβ3 Integrin Antibody in Human Hepatocellular Carcinoma HepG2 Cells
Source: PLoS One. 2016 Jul 8;11(7):e0158592. doi: 10.1371/journal.pone.0158592 (PMC4938599; doi:10.1371/journal.pone.0158592)

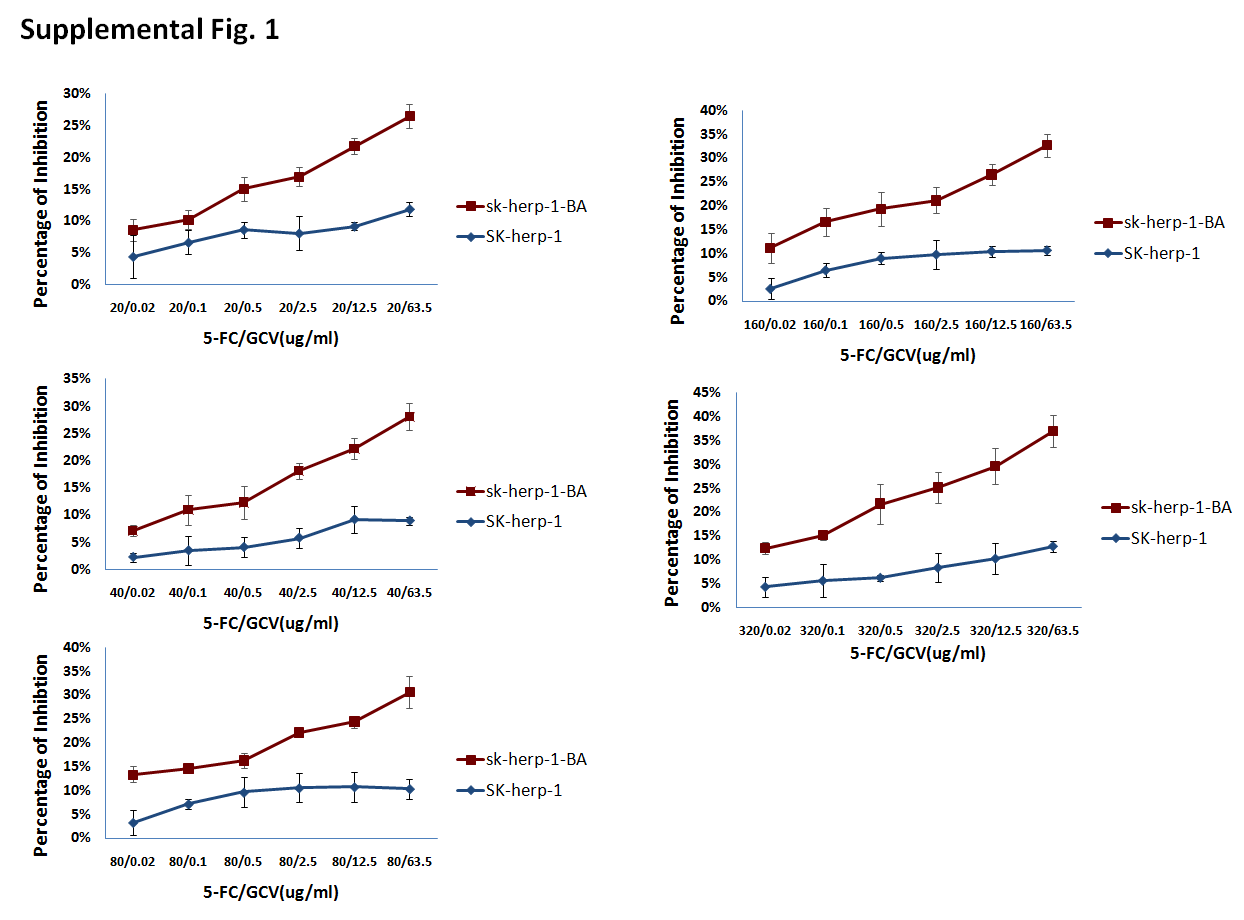

Supplement: S1 Fig — 5-FC / GCV with ultrasound plus CMBs was served as control (mean ± SD of three experiments; *p < 0.05). (JPG) [file pone.0158592.s001.jpg]

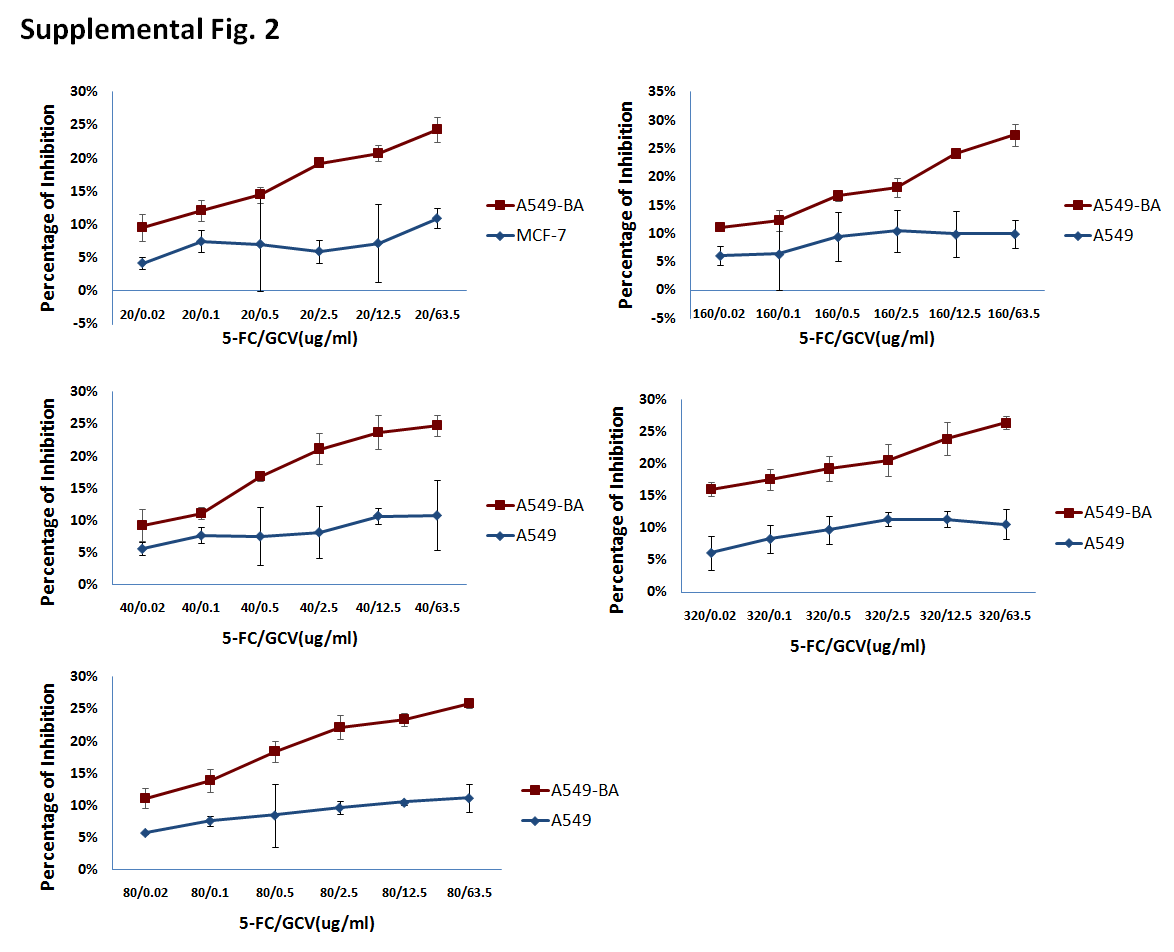

Supplement: S2 Fig — 5-FC / GCV with ultrasound plus CMBs was served as control (mean ± SD of three experiments; *p < 0.05). (JPG) [file pone.0158592.s002.jpg]
